# The complete chloroplast genome of *Epimedium jinchengshanense* Y. J. Zhang & J. Q. Li (Berberidaceae), an ornamental and medicinal species

**DOI:** 10.1080/23802359.2022.2044402

**Published:** 2022-02-27

**Authors:** Yanqin Xu, Limei Fu, Xiaofang Huang, Fengqin Li, Congying Wang, Shengfu Hu

**Affiliations:** College of Pharmacy, Jiangxi University of Chinese Medicine, Nanchang, People’s Republic of China

**Keywords:** *Epimedium jinchengshanense*, Berberidaceae, chloroplast, phylogenetic relationship

## Abstract

*Epimedium jinchengshanense* Y. J. Zhang & J. Q. Li 2014 is an important ornamental and medicinal herb, but of unclear taxonomy. In this study, the complete chloroplast genome of *E. jinchengshanense* was sequenced. The genome was 157,169 bp in length, with a large single-copy region of 88, 520 bp, a small single-copy region of 17,075 bp and 2 inverted repeat regions of 25, 787 bp. The genome consisted of 113 unique genes, including 79 protein-coding genes, 30 tRNA genes and 4 rRNA genes. The GC contents were 38.78%. Phylogenetic analysis showed a close relationship between *E. jinchengshanense* and *E*. *ilicifolium*, which was explained by the morphological similarity of flowers and leaves of the two species.

## 1. Background

*Epimedium* L., the largest genus of Berberidaceae, is an herbaceous perennial with great economic value (Stearn 2002; Ying et al. 2011; Xu et al. 2019). Among the 68 published *Epimedium* species, 58 (85.3%) are distributed in China, and 57 are endemic to China (Xu et al. 2020a). The taxonomy and phylogenetic relationship within *Epimedium* remained controversial due to the large morphological variations of the genus (Liu et al. 2017; Xu et al. 2019, 2020a,b; Guo et al. 2022). Although the taxonomy system described by Stearn (2002) was globally accepted, 13 new species have been published in the last two decades (Xu et al. 2020a). Among them, *Epimedium jinchengshanense* Y. J. Zhang & J. Q. Li 2014, named after its type locality (Jinchengshan, Sichuan, China), was firstly described by Zhang et al. (2014) due to its unique floral characteristics. The plant had been previously treated as *E. wushanense*. It was narrowly distributed in montane forests and thickets in northeastern Sichuan, at elevations of 600–1500 m (Zhang et al. 2014). Despite its narrow distribution, *E. jinchengshanense* has important medicinal and ornamental values because of its abundant medicinal components and large and showy flowers (Zhang et al. 2014; Jiang 2020). In this study, the complete chloroplast (cp) genome of *E. jinchengshanense* was sequenced and analyzed, which will facilitate the taxonomic research, resource protection and utilization of the valuable germplasm.

## 2. Methods

The sample of *E. jinchengshanense* was collected from the type locality, Jinchengshan, Yilong County, Sichuan Province, China (latitude 31.2100, longitude 109.9006). A specimen was deposited at the Herbaria of Jiangxi University of Chinese Medicine (JXCM; https://www.jxutcm.edu.cn/, Yanqin Xu, xuyanqin927@163.com) under the voucher number S. X. Liu & L. J. Liu 2016027. DNA was isolated from 0.3 g silica-dried leaf tissue using the CTAB protocol (Doyle and Doyle 1987). DNA libraries were prepared with an insert size of 350 bp using NEBNext Ultra DNA Library Prep Kit. Paired-end sequencing (150 bp reads) was performed on an Illumina NovaSeq 6000 platform (San Diego, CA) at the Novogene Co. Ltd. (Beijing, China). Finally, 5.83 G raw data was obtained with the number of raw reads 19,429,756. The cp genome was assembled using GetOrganelle v1.7.1 with default parameters (Jin et al. 2020), and the degree of coverage was 99.75%. The genes were annotated using GeSeq (Tillich et al., 2017) with *E. ilicifolium* (NC_044897) as a reference. The software Geneious primer (www.geneious.com) was used for further manual annotation with *E. ilicifolium* as a reference. The cp genome sequence of *E. jinchengshanense* was deposited in the GenBank database (MZ603799).

The phylogenetic relationship within *Epimedium* was reconstructed based on cp genomes of *E. jinchengshanense* and additional 14 *Epimedium* species previously reported, with *Vancouveria hexandra* (MH423073) as an out-group. Using PhyML v.3.0 (Stéphane et al. 2010), the maximum-likelihood (ML) tree was built with the best model of TVM + G and 1000 bootstraps.

## 3. Results

The complete chloroplast genome of *E. jinchengshanense* was 157,169 bp in length. It exhibited a typical quadripartite structure, including a large single-copy (LSC) region of 88,520 bp, a small single-copy (SSC) region of 17,075 bp and 2 inverted repeat regions (IRs) of 25,787 bp. The overall GC content was 37.78%, with 37.37%, 32.75%, and 43.18% for LSC, SSC and IR regions, respectively. The cp genome encoded 113 unique genes (79 protein-coding genes, 30 tRNA genes and 4 rRNA genes).

**Figure 1.**
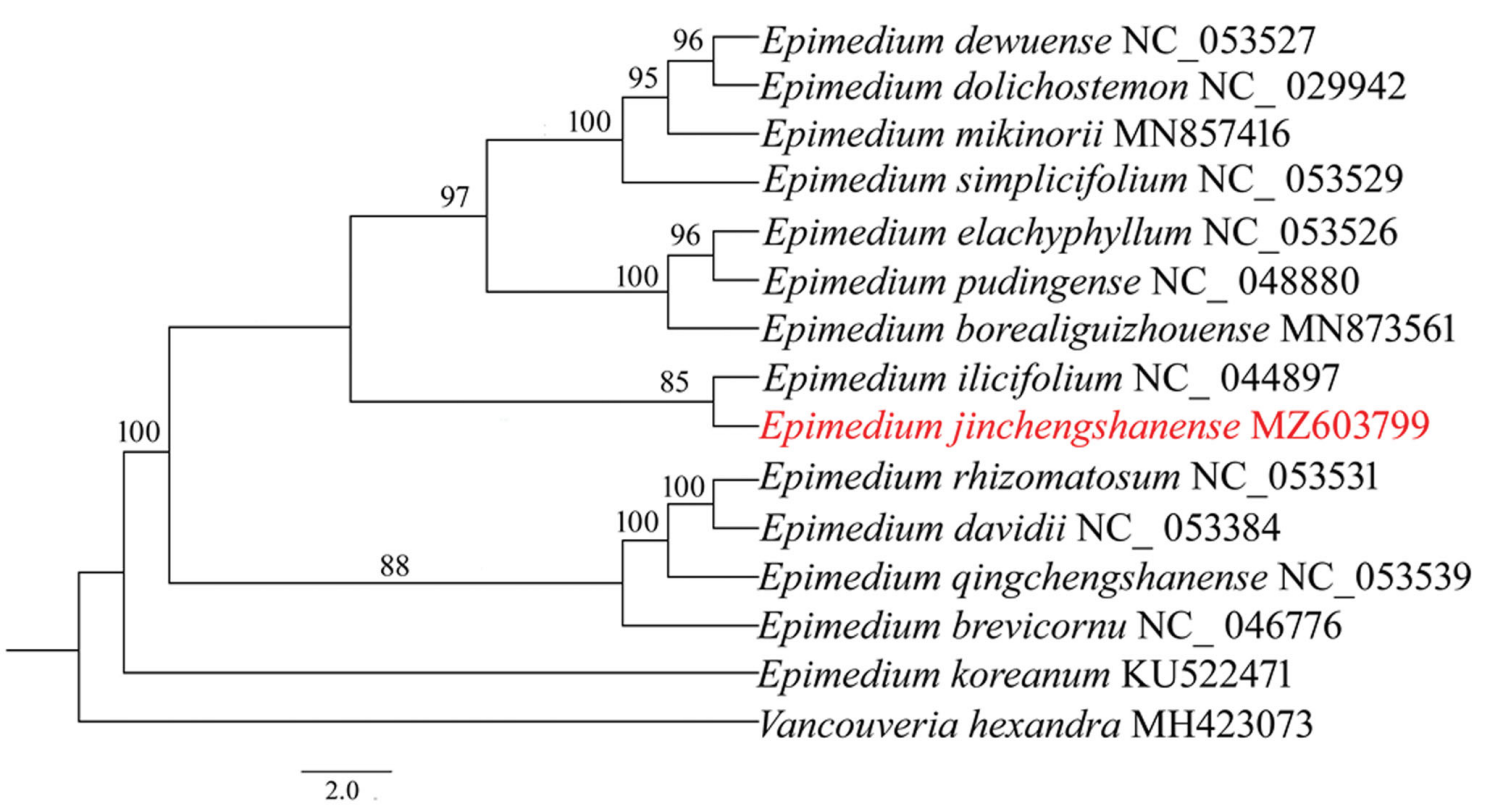
Maximum likelihood (ML) phylogenetic tree based on 15 complete chloroplast genomes, *Vancouveria hexandra* as an out-group. Numbers at nodes represented the bootstrap values.

Phylogenetic analysis showed that *E. jinchengshanense* clustered with *E*. *ilicifolium* (NC_044897) with a moderate bootstrap value 85% (Figure 1). The two *Epimedium* species were of morphological similarity of flowers and leaves, and they were distributed in the adjacent geographical areas, which might explain the close relationship between *E. jinchengshanense* and *E. ilicifolium*.

## Author contribution

Y. X. and S. H. were involved in the conception and design; L. F. and X. H. contributed the sample collection; F. L. and C. W. performed the analysis and interpretation of the data; Y. X., L. F. and C. W. contributed the drafting of the paper; Y. X. and S. H. revised it critically for intellectual content. All authors were involved in the final approval of the version to be published. All authors agree to be accountable for all aspects of the work.

## Data Availability

The genome sequence data that support the findings of this study are openly available in GenBank of NCBI at https://www.ncbi.nlm.nih.gov/nuccore/MZ603799.1/ under the accession no. MZ603799. The associated BioProject, SRA, and Bio-Sample numbers are PRJNA771137, SRR16347787, and SAMN22253056, respectively.
